# Spore liberation in mosses revisited

**DOI:** 10.1093/aobpla/plx075

**Published:** 2017-12-23

**Authors:** Friederike Gallenmüller, Max Langer, Simon Poppinga, Hanns-Heinz Kassemeyer, Thomas Speck

**Affiliations:** 1 Plant Biomechanics Group, Botanic Garden, University of Freiburg, Freiburg im Breisgau, Germany; 2 Freiburg Materials Research Center (FMF), Freiburg im Breisgau, Germany; 3 Department of Biology, State Institute of Viticulture and Enology, Freiburg im Breisgau, Germany; 4 Freiburg Centre for Interactive Materials and Bioinspired Technologies (FIT), Freiburg im Breisgau, Germany; 5 Competence Network Biomimetic, Freiburg im Breisgau, Germany

**Keywords:** *Brachythecium populeum*, Bryophyta, hygroscopic, moss, plant movement, spore dispersal

## Abstract

The ability to perform hygroscopic movements has evolved in many plant lineages and relates to a multitude of different functions such as seed burial, flower protection or regulation of diaspore release. In most mosses, spore release is controlled by hygroscopic movements of the peristome teeth and also of the spore capsule. Our study presents, for the first time, temporally and spatially well-resolved kinematic analyses of these complex shape changes in response to humidity conditions and provides insights into the sophisticated functional morphology and anatomy of the peristome teeth. In *Brachythecium populeum* the outer teeth of the peristome perform particularly complex hygroscopic movements during hydration and desiccation. Hydration induces fast inward dipping followed by partial re-straightening of the teeth. In their final shape, wet teeth close the capsule. During desiccation, the teeth perform an outward flicking followed by a re-straightening which opens the capsule. We present a kinematic analysis of these shape changes and of the underlying functional anatomy of the teeth. These teeth are shown to be composed of two layers which show longitudinal gradients in their material composition, structure and geometry. We hypothesize that these gradients result in (i) differences in swelling/shrinking capacity and velocity between the two layers composing the teeth, and in (ii) a gradient of velocity of swelling and shrinking from the tip to the base of the teeth. We propose these processes explain the observed movements regulating capsule opening or closing. This hypothesis is corroborated by experiments with isolated layers of peristome teeth. During hydration and desiccation, changes to the shape and mass of the whole spore capsule accompany the opening and closing. Results are discussed in relation to their significance for humidity-based regulation of spore release.

## Teaser

Gallenmüller et al. provide an analysis of spore liberation in mosses in response to humidity conditions. The study presents, for the first time, temporally and spatially well-resolved kinematic analyses of the complex hygroscopic movements performed by the peristome teeth inserted at the mouth of the spore capsule and gives insights into their sophisticated functional morphology and anatomy. The authors explain these movements by relating gradients in the composition of the peristome teeth to differences in swelling capacity and velocity between the tip and the base of the teeth and also between different teeth layers.

## Introduction

The ability to perform hygroscopic movements has evolved in a large variety of plant lineages and relates to a multitude of different functions. Examples of hygroscopic movement include seed burial brought about by coiling or bending movements of awns in *Erodium gruinum* and *Triticum turgidum* (Elbaum *et al.* 2007; Abraham *et al.* 2012; Abraham and Elbaum 2013a, b), the protection of flowers by bending movements of involucral bracts in *Syngonanthus elegans* (Oriani and Scatena 2009), the regulation of diaspore release by valve movements in ice plant seed capsules (folding and unfolding movements) (Harrington *et al.* 2011), the bending of pine cone seed scales (Harlow *et al.* 1964; Dawson *et al.* 1997; Poppinga *et al.* 2017), the movement of elaters in *Equisetum* spores (furling and unfurling movements) (Sitte 1963; Marmottant *et al.* 2013) and the movement of peristome teeth in moss sporophytes (flapping or bending movements) that regulate spore dispersal (Goebel 1895; Steinbrinck 1897; Patterson 1953; Ingold 1959; Schnepf *et al.* 1978).

Such hygroscopic movements in mosses are restricted to species with an arthrodontous peristome built up of portions of cell walls (in contrast to the nematodontous peristome type consisting entirely of dead cells). According to the origins of different peristomial layers, the arthrodontous peristomes can be divided into a haplolepidous type, which is characterized by a single ring of teeth, and the diplolepidous type with two rings of teeth arranged in an outer exostome and an inner endostome (see Ingold 1959; Vitt 1981; Shaw *et al.* 2011). After shedding of the operculum, which covers the peristome during maturation, peristome teeth bend outwards and, in xerocastique species, open the spore capsule when dry and bend inwards and close the capsule when wet. The reverse applies in hydrocastique species (Patterson 1953; Huttunen *et al.* 2004; Kungu *et al.* 2007). Such movements have been described for the single ring of teeth in species with a haplolepidous peristome as well as for the outer ring of teeth (the exostome) in mosses with a diplolepidous peristome (Ingold 1959; Schnepf *et al.* 1978; Vitt 1981; Shaw *et al.* 2011). Moreover, in some moss species, complex shape changes of the exostome teeth preceding the adoption of the final inwards or outwards bent shape have been described (Goebel 1895; Steinbrinck 1897; Ingold 1959).

**Figure 1.**
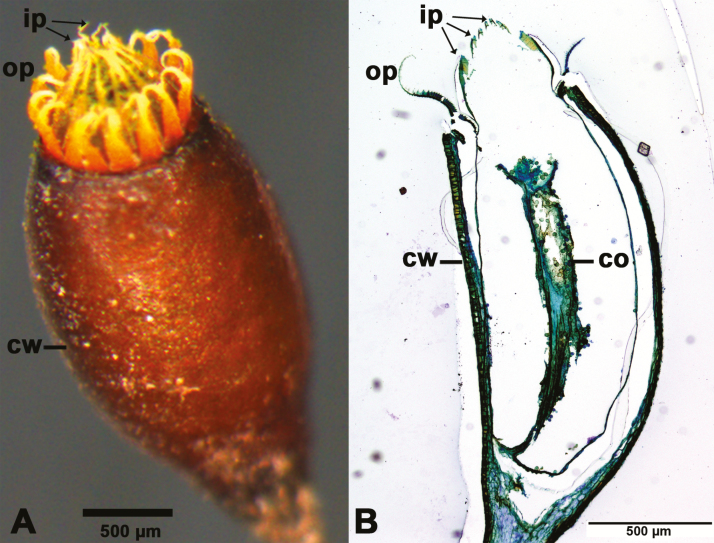
Mature spore capsule of *Brachythecium populeum* without operculum. (A) Whole capsule, (B) longitudinal section (thickness: 3 µm; staining: toluidine blue; embedding: Technovit). ip: inner peristome teeth (endostome); op: outer peristome teeth (exostome); cw: capsule wall; co: columella.

A first reference to peristome teeth functioning as veritable spore catapults during desiccation is provided by Goebel (1895), who interprets the extensions of the inner peristome teeth as catapulting organs in some diplolepidous Hypnaceae, Mniaceae and *Bryum* species. Steinbrinck (1897), Lazarenko (1957) and Ingold (1959) further describe an active spore discharge by oscillatory or flicking movements of the outer peristome teeth occurring during the transition between the dry and wet state in a variety of genera, including *Brachythecium* (Brachytheciaceae). These authors analyse the ontogenetic development and anatomy of the exostome in these species and conclude that differential velocity of water uptake and loss in the outer and inner layer of the outer peristome teeth is responsible for the observed movements. Lazarenko (1957) provides line drawings of the inwardly bent outer peristome teeth in wet and dry states (constituting the closed capsule states) and of the temporary outwardly bent shape at the transition between the dry and wet state (constituting the open capsule state) in *Timmia bavarica* (Timmiaceae). The exact nature and modes of function of these movements were not further elucidated in these studies. In the present work we analyse the functional morphology and kinematics of hygroscopic ‘flicking movements’ of the outer peristome teeth of *Brachythecium populeum*. The species is distributed in the temperate zones of the northern hemisphere and typically colonizes base-rich rocks, walls and tree bark (Nebel and Philippi 2001) (Fig. 1).

## Methods

### Plant material and experimental conditions

Samples of *B. populeum* bearing spore capsules were collected between February and June 2014 from stone walls in the Botanical Garden of the University of Freiburg and stored in transparent plastic boxes. During the experiments, room temperature was *T* = 25 ± 1 °C and the relative humidity was RH = 39.5 ± 2 % (*n* = 88, measured in steps of 30 min).

### Characterization of exostome teeth geometry

Lengths and widths of 29 exostome teeth cut from three different spore capsules of *B. populeum* were determined from images taken with a digital camera (ColorviewII; Olympus, Tokyo, Japan) mounted on a binocular stereomicroscope (SZX9; Olympus, Tokyo, Japan) using the ‘segmented line function’ of Fiji/ImageJ software (1.49a).

### Kinematical analyses of shape changes in exostome teeth and whole spore capsules

In a spore capsule, all inner peristome teeth and all but one of the outer peristome teeth were removed under a binocular stereomicroscope (SZX9; Olympus, Tokyo, Japan) using lockable tweezers and a scalpel. Videos (lateral views) of the hygroscopic movements of the remaining exostome tooth were recorded with the same stereomicroscope and a high-speed camera (Motion Pro Y4; IDT, Tallahassee, FL, USA) with a recording speed of 1000 fps. For the analysis of the movements of the exostome tooth, the storage of every seventh image of the resulting time series provided a suitable resolution. Spore capsules were held in place with lockable tweezers mounted on a micro-manipulator (114520; Leica Leitz, Wetzlar, Germany) and illuminated by an incident light source (Highlight 3100; Olympus, Tokyo, Japan). The spore capsule with the single remaining exostome tooth was hydrated by tilting the capsule into a Petri dish with distilled water and filmed in this position under water. Desiccation was filmed after removing the capsule from the water at room conditions as described above. The central axis of each recorded exostome tooth was retraced and tracked in every 50th image of the time series (equivalent to 2.8 fps) using Fiji/ImageJ software.

Spore release during desiccation of the peristome of an intact spore capsule (formerly hydrated with a water droplet was observed with the same high-speed camera (Motion Pro Y4; IDT, Tallahassee, FL, USA) with a recording speed of 1000 fps at room conditions as described above. Here, all recorded images were stored. Figure 9 presents a selection of images from a video.

In addition, a whole spore capsule was hydrated in distilled water in a Petri dish. Subsequent desiccation of the whole capsule took place under incident light (Highlight 3100; Olympus, Tokyo, Japan) and at room conditions. The observed hygroscopic shape changes of the capsule were filmed with a digital camera (Colorview II; Olympus, Tokyo, Japan) (recording speed: 0.1 fps) mounted on a binocular stereomicroscope (SZX9; Olympus, Tokyo, Japan). The diameter of the filmed capsule was measured every 20 s for the hydration-driven movements and every 80 s for the desiccation-driven motion(s) using ImageJ/Fiji software.

Additionally, the masses of 10 spore capsules with remaining opercula and of 10 capsules with opercula removed were determined at the dry state (after desiccation at room conditions) and at the fully hydrated state (after immersion in distilled water for 10 min).

### Analyses of tooth shape at different conditions of humidity

For analysing the shapes of the outer peristome teeth at different RH values, three spore capsules in which all but one of the exostome teeth were removed were glued to the lids of Petri dishes with adhesive film. Specific RH treatments were imposed inside closed Petri dishes using sulphuric acid solutions at concentrations ranging from 0 to 98.6 % according to Wilson (1921) and Oriani and Scatena (2009). Three Petri dishes were filled with 2.86 mL pure sulphuric acid, closed with the lids with one attached spore capsule each, and sealed with Parafilm. After a storage time of 24 h, images of the peristome teeth were taken through the lids. Subsequently, the solutions were replaced with a sulphuric acid solution at different concentrations and stored again for 24 h. This step was repeated to provide a total RH range from 0 to 98.6 % in 10 % steps. RH = 0 % was generated with pure sulphuric acid.

### Isolation of tooth layers

Inner and outer layer of an exostome tooth of *B. populeum* were separated using 400 mg pectinase (1.12 U mg^−1^; Sigma-Aldrich) dissolved in 8 mL distilled water and gum syrup according to Taylor (1959). Outer peristome teeth were excised and incubated in the pectinase solution in sealed Petri dishes for 3 days. Gum syrup was prepared by combining 25 mL of solution ‘A’ (40 g gum arabic, 0.5 g phenol crystals, 60 mL distilled water), 15 mL of solution ‘B’ (52 mL cane sugar in 30 mL distilled water) and 2 g glycerin. Syrup droplets were pipetted on a glass slide and dried overnight in room conditions. Before placing the peristome teeth on the gum (with either the inner or the outer layer facing the gum), the gum syrup was humidified using wet paper. The glass slides with the gum syrup and the peristome teeth were then dried in a drying cabinet for 1 h at 60 °C before the upper layer of the teeth was finally scratched away with a scalpel.

During preliminary experiments with isolated tooth layers placed directly into water, the tooth layers unavoidably moved out of the field of view of the binocular microscope. To avoid this, the isolated layers were, instead, positioned on filter paper. A water droplet was placed on the paper ~1 cm from the layers. The first contact of the isolated tooth layers with the spreading water droplet progressing through the paper was considered as start of the hydration process and the moment in which the water film retracted visibly from the isolated layers during evaporation as start of the desiccation process. During hydration, the isolated layers were covered entirely by water. Swelling and shrinking processes were recorded with a digital camera (ColorviewII; Olympus, Tokyo, Japan) (recording speed: 0.1 fps) mounted on a binocular stereomicroscope (SZX9; Olympus, Tokyo, Japan).

### Anatomy

Whole spore capsules were embedded in Technovit 7100 (Kulzer) (standard procedure) and cut with a rotary microtome (Leica, Wetzlar, Germany) (thickness 2–5 µm). Sections were dried at 60 °C in a drying cabinet for 12 h, stained with toluidine blue or FCA (fuchsin-chrysoidine-astra blue). Slides were sealed with Entellan (Merck). Images were taken with a digital camera (DP71; Olympus, Tokyo, Japan) mounted on a microscope (BX61; Olympus, Tokyo, Japan).

### Low-temperature scanning electron microscopy images

Low-temperature scanning electron microscopy (LT-SEM) images were prepared as described by Müller *et al.* (1991). A spore capsule without an operculum was cut and mounted on a specimen holder using a low-temperature mounting medium. After cryofixation in liquid nitrogen at −180 °C in a nitrogen cold stage (Gatan, Pleasanton, CA, USA), samples were transferred under a nitrogen atmosphere into a Cryopreparation unit (Alto 2500; Gatan, Pleasanton, CA, USA) attached to a SEM (XL30 ESEM Field Emission-Scanning-Electron-Microscope; Philips, Amsterdam, Netherlands). Ice crystals on the surface of the specimen were allowed to sublimate from the surface by raising the temperature to −80 °C for about 10 min. The specimens were sputter-coated with gold (20 nm) in an argon atmosphere and transferred into the SEM under high-vacuum conditions. The samples were observed at a stage temperature of −165 °C, using an acceleration voltage between 5 and 25 kV.

### Statistical analyses

Statistical analyses were carried out using R statistical software 3.4.0 (R Core Team 2017) with a significance level of α = 0.05. The ‘car’ and ‘stats’ packages (Fox and Weisberg 2011; R Core Team 2017) were used. All data sets were tested for normality by the Shapiro–Wilk test (‘shapiro.test’ function). Median and interquartile ranges were calculated for not normally distributed data (temperature and relative air humidity data). Mean and standard deviation were computed for normally distributed data (teeth geometry and capsule weight data). Capsule weight data were further tested for homoscedasticity by Levene’s test (‘leveneTest’ function). Paired *t*-tests (‘t.test’ function) were performed to test the influence of the presence of the operculum on water uptake. Unpaired *t*-tests were used to compare the weight of wet capsules with and without operculum (homoscedastic) and dry capsules with and without operculum, respectively (heteroscedastic).

## Results

**Figure 2.**
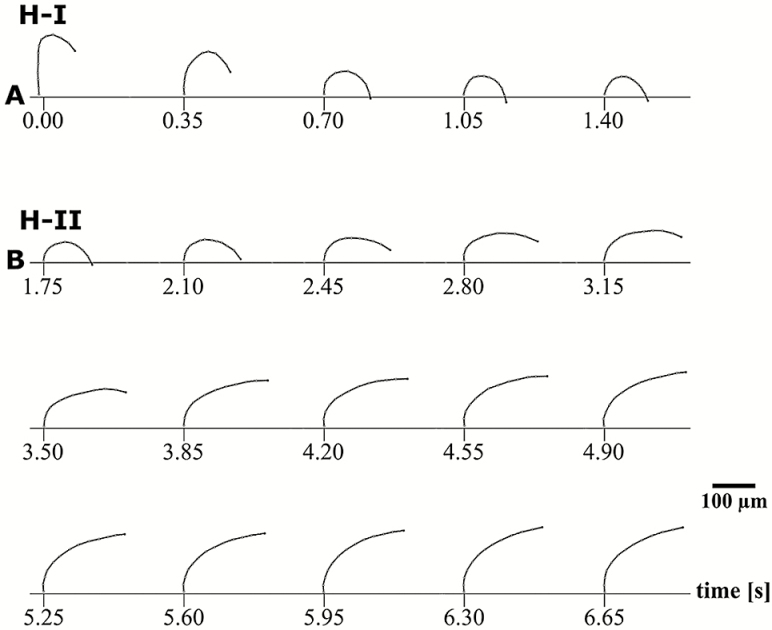
Movement of an initially dry, single outer peristome tooth of *Brachythecium populeum* during immersion in water. (A) Hydration phase I = phase H-I, inward movement (starting from a ‘hook-like’ shape), (B) hydration phase II = phase H-II, partial re-straightening with a remaining curvature below the middle region of the tooth. The interior of the spore capsule is located on the right side of the tooth, the exterior on its left side **[see Supporting Information—Video 1]**.

**Figure 3.**
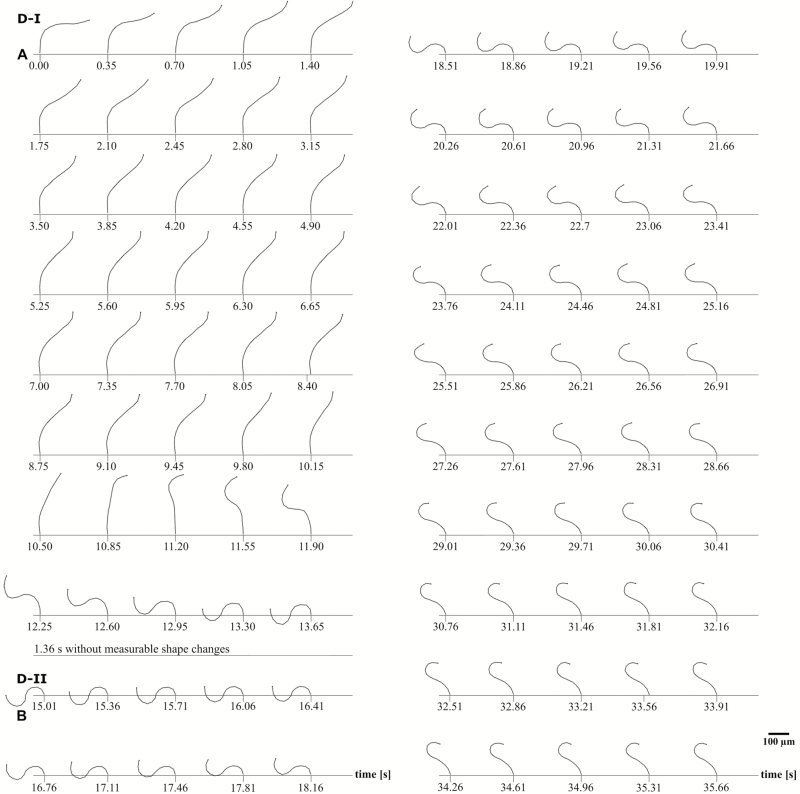
Movement of a single outer peristome tooth of *Brachythecium populeum* during desiccation. (A) Desiccation phase I = phase D-I, opening movement with form change into a ‘S-shaped’ form, (B) desiccation phase II = phase D-II, restoration of a more upright position and change into a ‘hook-like’ shape typical for the dry state (see Fig. 2). The interior of the spore capsule is located on the right side of the tooth, the exterior on its left side **[see Supporting Information—Video 2A, 2B]**.

**Figure 4.**
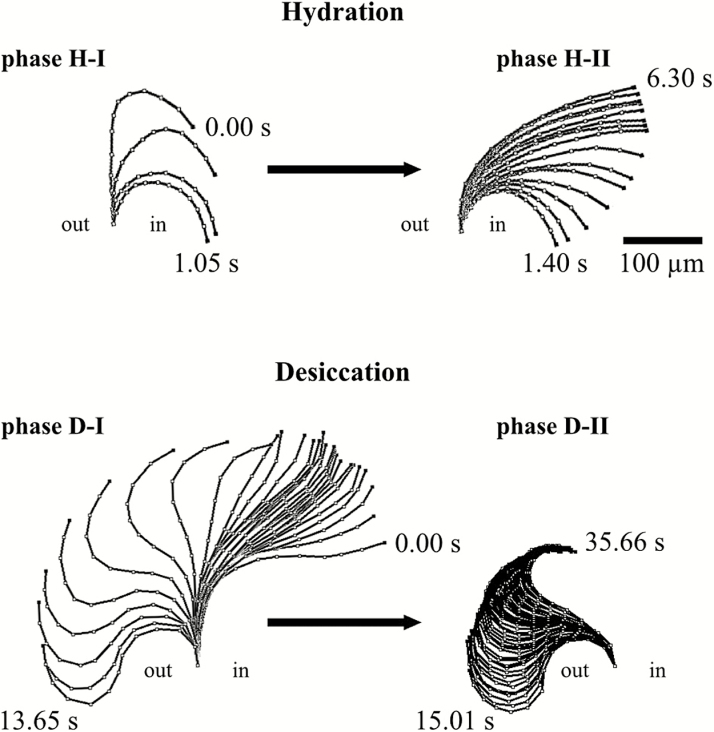
Illustration summarizing the hygroscopic movement of a single outer peristome tooth of *Brachythecium populeum* observed during hydration (phases H-I and H-II) and desiccation (phases D-I and D-II). ‘in’ designates the inside of the spore capsule, ‘out’ the outside.

In *B. populeum*, the outer peristome teeth of the spore capsules perform complex movements during both hydration and desiccation and adopt different final shapes at the end of these movements in dry and wet capsules, respectively (Figs 2–4) **[see Supporting Information—Video 1, 2A, 2B]**. The inner peristome teeth do not show hygroscopic movements or shape changes and remain in an inwardly bent position, forming a permanent dome over the capsule mouth (Fig. 1).

### Characterization of exostome teeth geometry

The mean length of the tapered, outer peristome teeth is 306 ± 35 µm (*n* = 22). The mean width at the basal region is 87 ± 13 µm (*n* = 27), 57 ± 10 µm (*n* = 27) at the middle region, and 15 ± 4 µm 10 µm below the tip (*n* = 26).

### Kinematical analyses of shape changes in exostome teeth

In dry capsules, the exostome teeth adopt an upright position with straight lower and middle sections and typically inward curved tips, which give a ‘hook-like’ shape (Fig. 2A at 0.00 s). When a dry capsule is immersed in water, the outer peristome teeth perform a fast inward bending due to inward curvature which starts at the tip and then progresses to the middle and basal region of the teeth, thereby dipping between the alternating inner peristome teeth of the endostome (hydration phase I = H-I, duration ~1.4 s in our experiment with a single tooth) (Fig. 2A). This inward movement is followed immediately by a partial re-straightening of the middle section of the exostome teeth, leading to a final shape with a wide curvature in the basal region of the teeth and thus to closure of the capsule mouth (hydration phase II = H-II, duration ~4.9 s) (Fig. 2B). During desiccation of formerly fully hydrated capsules, the outer peristome teeth perform a fast outward movement, thereby gaining a final S-shape (desiccation phase I = D-I, duration in total 13.65 s). The movement begins with a straightening of the basal region (duration ~10.8 s) followed by an inward curvature of the apical half and, almost simultaneously, a commencement of deformation by the basal half into an outward curvature (duration ~2.8 s). This last movement causes the tooth to ‘flick outwards’ (Fig. 3A). This outward movement is followed by a re-straightening of the basal section of the teeth and a progression of the inward curvature from the middle to the apical section, resulting in the typical upright ‘opening position’ with tips in the inward curved ‘hook-like’ shape observed in dry spore capsules (desiccation phase II = D-II) (in total 20.65 s; Figs 1, 3B and 4).

### Analyses of tooth shape at different conditions of humidity

**Figure 5.**
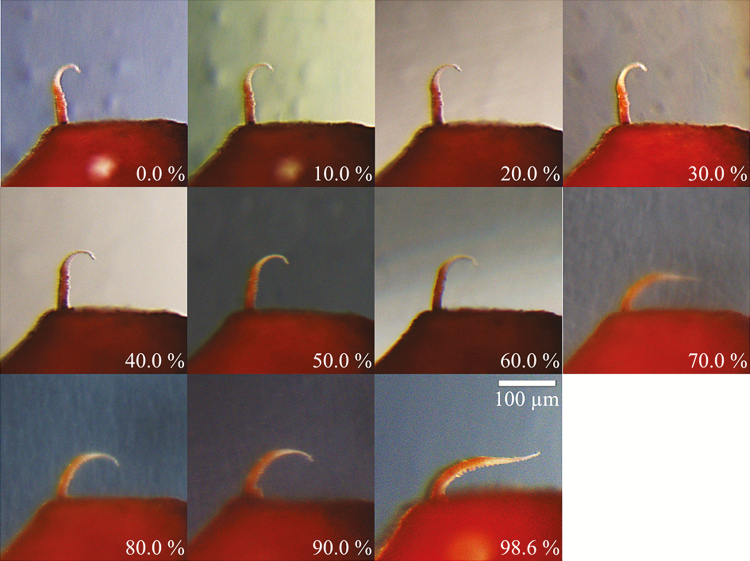
Capsule with a single outer peristome tooth exposed to different levels of relative air humidity. The other outer peristome teeth were removed in order to provide a better visibility of the tooth shape and orientation. Shapes of two other outer peristome teeth submitted to the same conditions were very similar (data not shown).

The observations of shape changes in the outer peristomal teeth during hydration and desiccation processes are consistent with observations made on capsules submitted to different levels of RH (Fig. 5). In the RH range between 0–10 % and 50–60 %, the outer peristome teeth adopt the orientation and the ‘hook-like’ shape typical of dry teeth in the ‘opening position’. With RH exceeding 60–70 %, a change into a shape with a wide curvature in the basal section is observed, comparable to the shape adopted by fully hydrated peristome teeth of capsules immersed into water in the ‘closing position’.

### Hygroscopic shape changes of spore capsules

**Figure 6.**
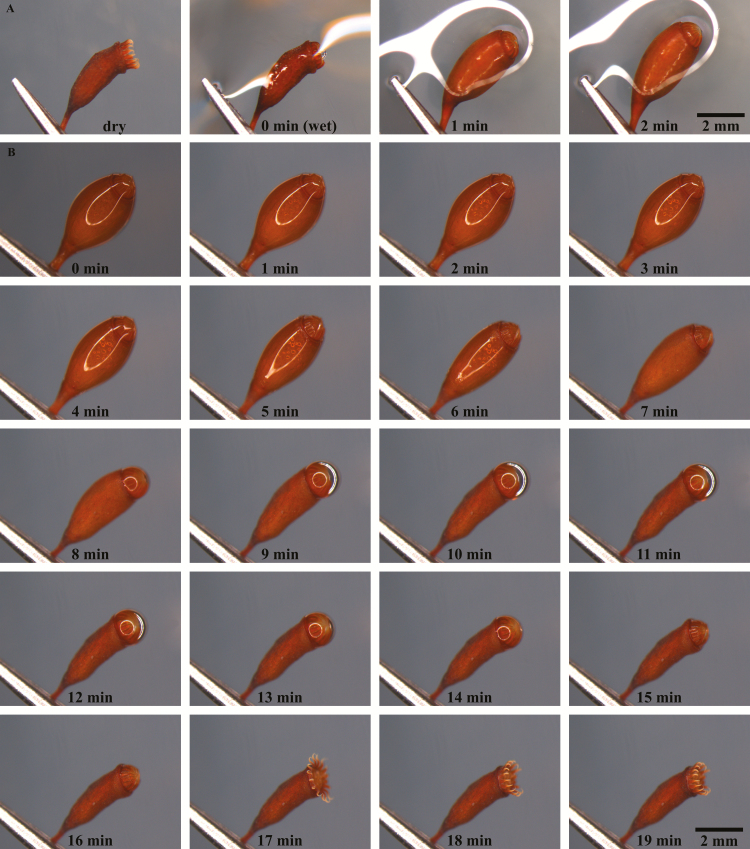
Hygroscopic shape change of a spore capsule of *Brachythecium populeum*. (A) Hydration by immersion in water (min 1) with volume increase (outward bulging of the capsule walls) and erection of the capsule, total duration of the swelling process 10 min, (B) desiccation at *T* = 25 ± 1 °C and RH = 39.5 ± 2 % with volume reduction and inward buckling of the capsule accompanied by emersion of a water droplet squeezed through the peristome (min 8 and min 9); then evaporation of the water droplet, and typical outward flicking movement of the outer peristome teeth during desiccation (temporary outwards bent shape visible at min 17) **[see Supporting Information—Video 3A, 3B]**.

In addition to the fast hygroscopic movements of the outer peristome teeth, slower changes in the shape of the whole spore capsule take place during hydration and desiccation (Fig. 6) **[see Supporting Information—Video 3A, 3B]**. A dry capsule is approximately cylindrical, tapering towards the seta in the basal region (the apophysis), slightly bent in the upper section and constricted below the peristome. Hydration of a dry capsule by immersion in water increases capsule volume accompanied by erection of the capsule and an outward bulging of the capsule walls (Fig. 6A, total duration in single experiment: 10 min). During subsequent desiccation, the capsule wall contracts inwards and the capsule returns to its initial shape typically adopted when dry. In the course of shrinkage a water droplet is squeezed out of the capsule, through the inner peristome teeth and through the (at this moment still inwardly bent) outer peristome teeth. The typical outward/inward flicking movement of the outer peristome teeth during desiccation takes place only after evaporation of the water droplet. At this point, the constriction of the spore capsule below its mouth is nearly complete (Fig. 6B, total duration in our experiment 17 min at *T* = 25 ± 1 °C and RH = 39.5 ± 2 %). Inflation of the capsule during hydration entails a slight inward tilting of the capsule rim, thus contributing to the final ‘closing’ position of the outer peristome teeth in fully hydrated state or when exposed to a RH of more than 60 % (Figs 5 and 6A). Movement patterns of the outer teeth in the intact peristome of the capsule are identical to those observed in an isolated single peristome tooth (Figs 4 and 6).

**Figure 7.**
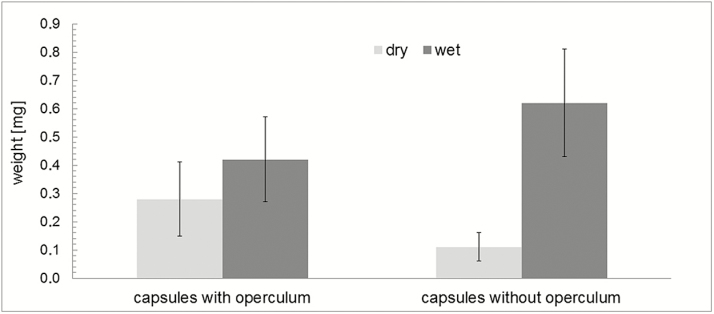
Mass differences (mean values and standard deviations) of dry and wet spore capsules and comparison of closed capsules (with the operculum still attached) and open capsules (without operculum). Wet capsules were measured after immersion in water, *n* = 10 in each set. Wet capsules have a significantly higher mass than dry capsules (paired *t*-test, *t*(9) = −5.33, *P* = 4.73×10^−4^ for capsules with operculum; paired *t*-test, *t*(9) = −9.98, *P* = 3.64×10^−6^ for open capsules). Dry capsules without operculum have significantly lower masses than dry capsules with operculum (unpaired *t*-test, *t*(11.47) = 3.89, *P* = 2.33×10^−3^), whereas—due to the better penetration of water into the capsules—wet capsules without operculum have significantly higher masses than wet capsules with operculum (unpaired *t*-test, *t*(18) = −2.55, *P* = 1.99×10^−2^).

**Figure 8.**
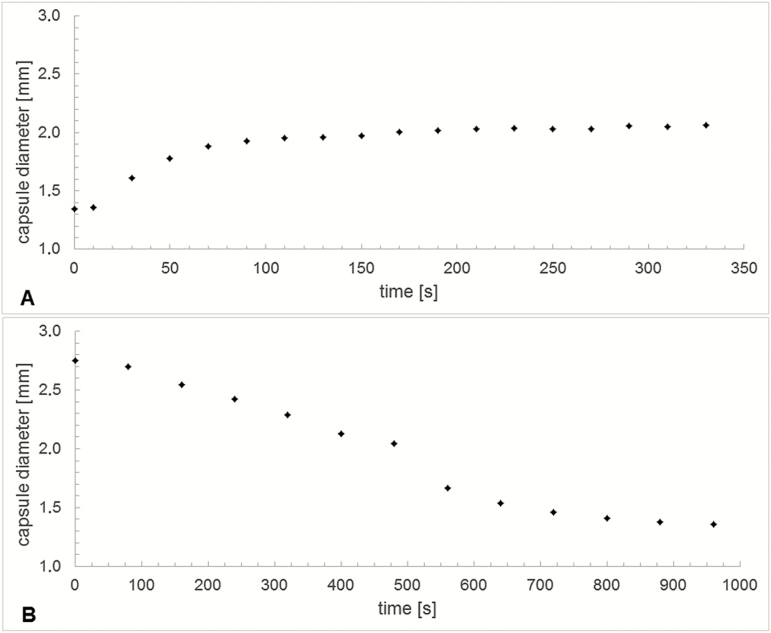
Variation of capsule diameter measured at the thickest middle region over time during (A) hydration and (B) desiccation, respectively.

**Figure 9.**
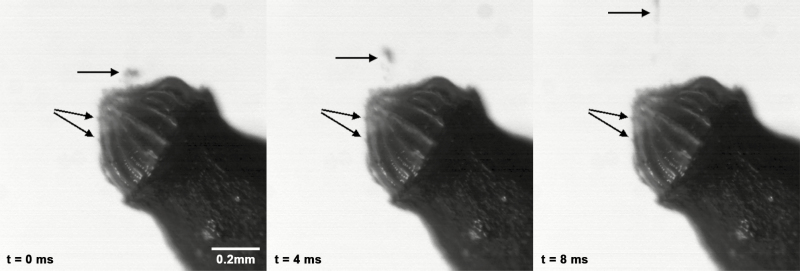
Spores (arrows) sifting through the peristome and clinging at the peristome teeth of a spore capsule of *Brachythecium populeum* (images selected from a high-speed video taken at a time distance of 4 ms, when peristome teeth start to open during desiccation). The capsule itself was not hydrated **[see Supporting Information—Video 4]**.

Water uptake during hydration (measured as mass gained) is significantly higher in capsules in which the operculum is removed (546 ± 172 %) than in capsules with an operculum still attached (150 ± 55 %) (Fig. 7). The outer diameter of the capsule (measured at the thickest part in the middle region) also changes considerably during the hydration and desiccation process (Fig. 8). At the transition between the wet and the dry state and in addition to the spore release accompanying the hygroscopic outward ‘flicking’ movements of the peristome teeth, we have also observed spores simply sifting through the inner and outer peristome teeth. This starts once a small degree of opening of the exostome takes place during the first milliseconds of phase I desiccation (D-I) (Fig. 9) **[see Supporting Information—Video 4]**.

### Analyses of anatomy and LT-SEM images

**Figure 10.**
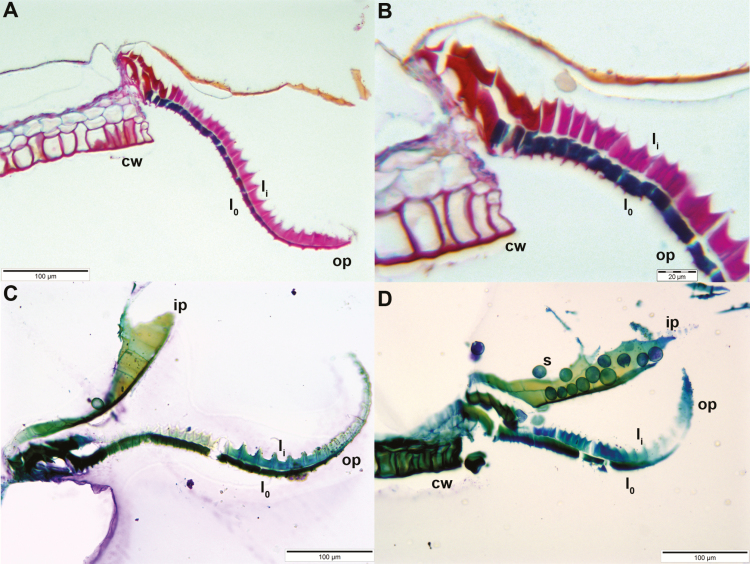
Longitudinal sections of *Brachythecium populeum* peristome teeth (thickness: 3 µm; staining: (A) and (B) FCA, (C) and (D) toluidine blue; embedding: Technovit). The different staining of the outer layer l_o_ (blue by FCA/dark green by toluidine blue) and the inner layer l_i_ (pink by FCA/light blue or green by toluidine blue) visualizes the two-ply structure of outer peristome teeth (op). The articulation-like structure at the insertion of the tooth at the capsule rim is also composed of two layers but they stain in a different colour than the outer and inner layer of the peristome tooth itself (red in FCA staining: A and B). cw: capsule wall. In (D) spores (s) are clinging to an inner peristome tooth (ip).

**Figure 11.**
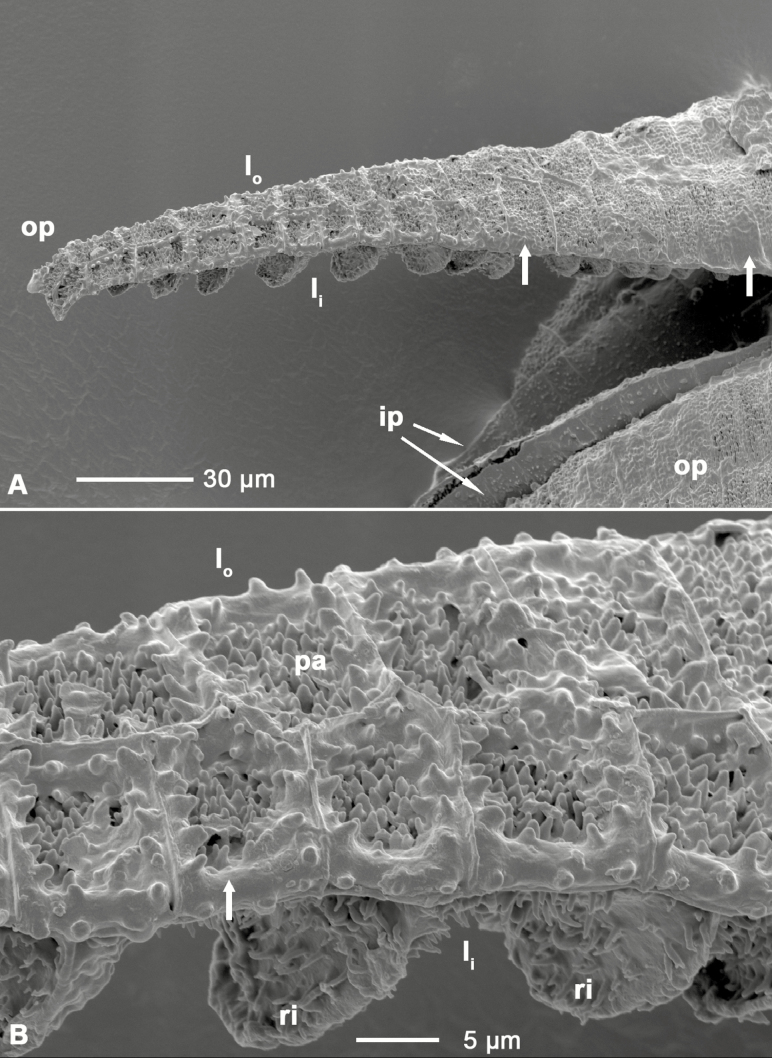
(A) LT-SEM image of outer and inner peristome teeth of *Brachythecium populeum*; (B) close up of the middle region of the outer peristome tooth shown in (A). op: outer peristome tooth; ip: inner peristome tooth; l_o_: outer layer of the outer peristome tooth; l_i_: inner layer with partly visible ridges; ri: ridges; pa: papillae. Broad arrows: smooth substance coating the papillae and segment borders, with an increasing gradient of coated area from the base to the tip of the outer peristome teeth.

Longitudinal sections of the outer peristome teeth reveal a structural organization with two layers differing in their material composition and morphology. Both FCA and toluidine blue stain the inner and outer layer differently (Fig. 10A). In the outer layer, both the thickness and the intensity of FCA staining gradually increase from the tip to the base of the tooth (Fig. 10A). The inner layer is composed of segments bordered by ridges which are up to 10 µm high, whereas smaller protruding ribs of ~3–4 µm height are apparent on the segments of the outer layer. The length of the segments decreases gradually from the tip to the base of the teeth (Figs 10 and 11). The articulation-like basal region connecting the outer peristome teeth to the rim of the capsule is also composed of two layers but stained in a different colour than the outer and inner layer of the peristome tooth itself, indicating a different material composition. Low-temperature scanning electron microscopy images reveal that the surface of the cell wall segments building the outer peristome teeth is densely covered by small cylindrical or cone-shaped papillae on both the inner and outer layer (Fig. 11). These structures are partly coated by a smooth film of an unknown substance. The coated area decreases gradually from the base to the tip of a tooth (Fig. 11). The non-hygroscopic inner peristome teeth display approximately smooth surfaces in LT-SEM (Fig. 11).

**Figure 12.**
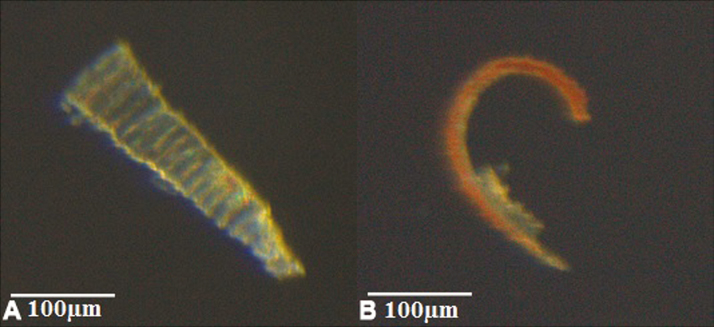
Isolated inner layer (A) and outer layer (B) of an outer peristome tooth of *Brachythecium populeum*, immersed in water. Due to the unavoidable destruction of one layer during preparation the depicted layers originate from two different teeth. The outer layer bears remnants of the inner layer at the apical part (arrow). The inner layer has adopted a straight shape, whereas the outer layer is bent inward (bending direction given in relation to the original arrangement of the tooth on the capsule) **[see Supporting Information—Video 5A, 5B]**.

When isolated, the inner and outer layer of an outer peristome tooth show different movements and finally adopt different shapes. The inner layer extends into a straight shape, whereas the outer layer curves inwards in its basal and middle section (Fig. 12) (bending direction given in relation to the original arrangement of the tooth on the capsule). This curvature increases slightly, when the outer layer is immersed into water. We cannot also exclude the possibility that the basal region of the outer layer principally undergoes bending deformation during hydration. This uncertainty arises because it was not possible to remove the inner layer completely in our experiment (Fig. 12).

**Figure 13.**
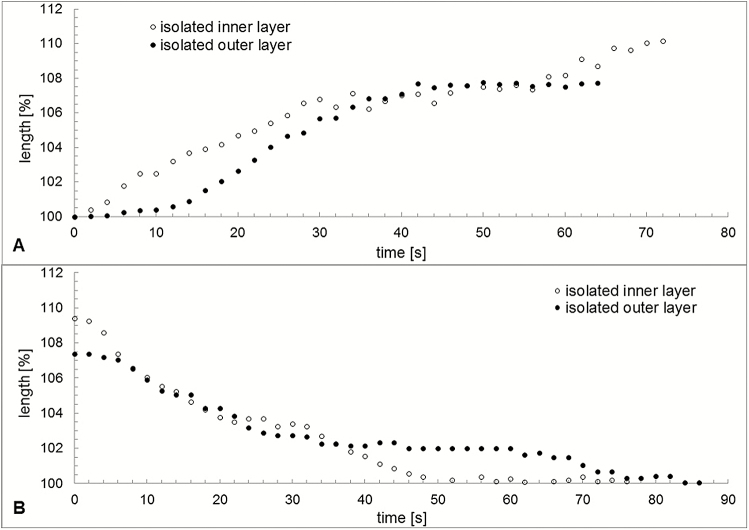
Variation in length over time during hydration (A) and desiccation (B) of an isolated outer and an isolated inner layer of an outer peristome tooth of *Brachythecium populeum*. Due to the unavoidable destruction of one layer during preparation the two layers originate from two different peristome teeth. *t* = 0 corresponds to the contact with water in (A) and the disappearance of the water film in (B), as visible in the light microscope.

Swelling and shrinking of an isolated inner and outer layer of exostome teeth, measured as percentage change in length, differ both in capacity and rate during hydration and desiccation (Fig. 13) **[see Supporting Information—Video 5A, 5B]**. Water uptake results in a longitudinal swelling up to 107.7 % of the outer layer and to 110.2 % of the inner layer. During hydration, at first the inner layer swells faster, then the outer layer. Both hydration curves saturate after ~40–50 s. Conversely, the inner layer at first shrinks faster and then slower during desiccation after evaporation of the water (Fig. 13).

## Discussion

Due to the different shapes adopted by the outer peristome teeth in the dry and wet states, the spore capsules of *B. populeum* are tightly closed when wet and partly opened when dry. Johansson *et al.* (2016) have shown that in *Brachythecium rutabulum* the inwards bent position of outer peristome teeth at the wet state effectively prevents spore release. The authors analysed the positions of the exostome teeth and spore release under different conditions of RH. They found a RH threshold of 69.3 % for the closure of the outer peristome during increase of RH and a threshold of 85.5 % for opening during a decrease of RH. The authors further distinguished between an ‘open state’ at 40 % RH, a ‘closing state’ at 75 % RH and a ‘closed state’ at 90 % RH and showed corresponding images of *B. rutabulum*, a species in which the exostome teeth adopt shapes comparable to the final shapes of outer peristome teeth reported in our study for *B. populeum* at the wet and dry state. Accordingly, it can be assumed that the closure of the outer peristome teeth at the wet state effectively prevents the spore release also in *B. populeum.* This assumption is corroborated by the fact that we never observed spore release by fully hydrated spore capsules. Such a closure and opening of the structure containing the dispersal units according to conditions of humidity has been described for a variety of plant species, i.e. by Harlow *et al.* (1964) for pine cones, by Harrington *et al.* (2011) for ice plant capsules and by Goebel (1895) for different moss species. The consequential restriction of the release of dispersal units at dry (or wet) conditions is commonly interpreted as a selective advantage in species with wind-dispersed seeds or spores. Their dispersal units are supposed to stay airborne for longer and thus cover longer distances when the air is dry than under rain or fog (Huttunen *et al.* 2004; Jongejans *et al.* 2007).

In moss genera with arthrodontous and diplolepidous peristomes such as those of *Brachythecium*, the two-ply structure (functional bilayer setup) of the outer peristome teeth is a result of its ontogenetic formation by two adjacent cell layers in the maturing spore capsule. The cells of the so-called primary peristomial layer deposit the cell wall material which builds up the inner layer of a tooth while the adjacent cells of the so-called outer peristomial layer deposit the cell wall material to build the outer layer of this tooth on the other side of the middle lamella. The final tooth is then formed by autolysis of the adjacent peristomial layers and of periclinal walls between adjacent teeth during maturation of the sporophyte (Ingold 1959; Kreulen 1972; Kungu *et al.* 2007; Shaw *et al.* 2011). The difference of shape adopted by dry and wet outer peristome teeth can be interpreted as a consequence of different swelling capacities of the two layers composing the teeth (being built by different types of cells). The same mechanism has been hypothesized for the different layers found in hygroscopic pine cone scales (Harlow *et al.* 1964; Dawson *et al.* 1997; Reyssat and Mahadevan 2009; Poppinga *et al.* 2017), valves of ice plant capsules (Harrington *et al.* 2011), wheat awns (Elbaum *et al.* 2008), involucral bracts of *Syngonanthus elegans* (Oriani and Scatena 2009) and peristome teeth of other moss species (Steinbrinck 1897; Ingold 1959). So far, the adoption of different shapes in the wet and dry states observed in the outer peristome teeth of *B. populeum* can be classified as yet another typical example of hygroscopic movements based on a bilayer structure. The higher capacity of swelling of the inner layer found in our study is consistent with the observed straightening of the middle and upper section of the exostome teeth during hydration and may—along with the observed curvature of an isolated outer layer—explain the different final shapes of wet and dry exostome teeth.

In contrast, the hygroscopic movements occurring at the transition between the wet and dry states and preceding the formation of the final shapes of the outer peristome teeth fundamentally differ from other hygroscopic movements of plant structures described so far. This is because they can be attributed not only to differences in swelling capacity but also to additional differences in swelling (and shrinking) velocity of the two layers building the structure. This has already been suspected by Ingold (1959) and Steinbrinck (1897) for the exostomes of different moss genera for which the authors mention movements similar to those described here. The theory of differences in swelling (and shrinking) velocity being the functional principle underlying the observed complex movement patterns attracts further support from the differences of velocity of swelling and shrinking by isolated inner and outer layers of exostome teeth found in our experiments. However, the findings should be interpreted with caution, as the observed variation in length of the isolated layers cannot be simply transferred to the situation in intact teeth. They also do not cover the most relevant first seconds (in which the movements of intact teeth take place) with a sufficiently high resolution in time.

We further hypothesize that the velocity of water uptake and loss not only differs between the outer and inner layer, but additionally follows a gradient from the tip to the base of the outer peristome teeth of *B. populeum*. Four gradients in structure and material composition observed in these teeth may underlie such a gradient of swelling or shrinking velocity. From the tip to base of an exostome tooth (i) its width, (ii) its thickness, (iii) the area of coverage of the papillae by the smooth substance revealed in LT-SEM on the outer surface and (iv) the material density (apparent in staining intensity) all gradually increase. Assuming that these gradients result in a gradient of swelling velocity and that the swelling velocity is in general higher in the outer layer of the tooth than in its inner layer, the observed hygroscopic movements can be interpreted as follows. The outer layer of an exostome tooth swells faster than the inner layer, with an increasing gradient of velocity from the tip to the base. This corresponds to the progression of the inward curvature from the tip to the base of the tooth during hydration, causing its inward dipping between the endostome teeth (hydration phase I). The slower swelling of the inner layer then results in the partial re-straightening of the tooth and ends with the adoption of its final shape at the ‘closing position’ (hydration phase II). Conversely, the overlay of (i) different shrinking capacities of the outer and inner layer, (ii) different shrinking velocities in the inner and outer layer and (iii) a shrinking velocity gradient from the tip to the base of the tooth during desiccation causes the outward flicking movement, accompanied by the adoption of an S-shape (desiccation phase I), whereas the slower shrinking of the inner layer results in the re-straightening of the tooth and ends with the adoption of its final ‘hook-like’ shape in the opening position. The intrinsic shape differences observed between isolated inner and outer exostome tooth layers possibly also influence the movement of intact exostome teeth and their final shapes in the wet and dry state.

Additionally, the closure and opening of the capsule mouth by the outer peristome teeth is further enhanced by the shape changes of the whole spore capsule, causing its rim with the attached peristome teeth to tilt inwards during hydration and to straighten again when drying.

We also hypothesize that, at each hydration and subsequent desiccation process, a portion of spores gets transported from the interior of the capsule to the outside by the peristome teeth and exposed to the ambient air. Here, two complementary mechanisms can be pictured. The fast inward dipping of the outer peristome teeth between the inner teeth observed during capsule hydration can be interpreted as a ‘scooping’ mechanism resulting in an upwards transport of spores, which are located between or under the inner peristome teeth. The (albeit relatively slow) following outward movement during desiccation can be seen as a slow catapulting mechanism flicking the spores clinging to the teeth structures into the air. This is consistent with the findings of Ingold (1959) who describes a similar fast outward swinging of the outer peristome teeth at the transition from the wet to the dry state in *Eurhynchium confertum* (Brachytheciaceae) and observed a repeated ‘violent discharge’ of spores during this process. However, considering the morphology of the capsule, Ingold (1959) stated that ‘it is difficult to see how more than 5 or 10 % of the spores could be scooped out by the teeth’. Here, a second mechanism may come into effect. The water droplet ‘squeezed’ through the dome of the endostome and the—at this point still fully inwards bent—exostome teeth taking place during desiccation may contain and bring up further spores from the interior of the capsule that attach to the inner and/or outer peristome teeth during the process. This spore transport process may be further enhanced by constriction of the capsule. The measured masses of wet and dry capsules of *B. populeum* with and without operculum show that water actually penetrates mature capsules without an operculum when they are immersed in water. Observations of spores clinging to the indentations of the inner layer of the outer peristome teeth further support this interpretation. In addition to the restriction of spore release to dry conditions favourable for long distance wind dispersal (when the exostome teeth have adopted the ‘opening position’), this ‘flicking mechanism’ results in a release of a portion of spores at each transition of the wet to the dry state. Lazarenko (1957) studied the particular hygroscopic movements of peristome teeth in *Timmia* (Timmiaceae), which also occur at the transition of the wet to the dry state and stated that they can be considered as an ‘apparatus for the ejection of spores’. Lazarenko (1957) further concluded that such movements of exostome teeth result in a spore release directly after rain or dew, and therefore under conditions of sufficient moisture for the germination of the spores. The spore release in *B. populeum*, as analysed in the present study, apparently combines both strategies: an ejection of spores directly after rain (when substrates in direct vicinity are wet), probably beneficial for population maintenance, and a continuous sifting out of spores under ongoing dry conditions (when the wind-distributed spores are thought to cover long distances), probably beneficial for gene flow to other populations and for colonization of new habitats.

The exact nature of the ecological significance of the ‘flicking mechanism’ remains to be elucidated. Several characters of the moss sporophyte have been considered as being an adaptation to particular ecological conditions. For example, Vitt (1981) discusses the combination of erect capsules, short seta and a reduced peristome as typical for species adapted to xerophytic habitats. In order to evaluate the ecological significance of the described hygroscopic mechanism a record of its presence in different other moss species with an arthrodontous and diplolepidous peristome is needed, along with an analysis of the surface structure of their exostome teeth, which can vary considerably within a family (Kungu *et al.* 2007) or even within a genus (Allen 1980). Further studies focusing on the adhesive properties of the spores to wet and dry surface structures of the peristome teeth and an identification of the material building the papillose structures and the coating substance on the surfaces of the exostome teeth will help to understand better the relationship between functional morphology and the functional principles of the opening and closing mechanism of the spore capsule.

## Conclusions

Spore release in the moss *B. populeum* is controlled by complex hygroscopic movements of the outer peristome teeth and the spore capsule. Due to the different shapes adopted by the outer peristome teeth in the dry and wet state, the spore capsules of *B. populeum* are tightly closed when wet and partly opened when dry. Additionally, at the transition from the dry to the wet state a fast inward dipping followed by a partial re-straightening of the outer peristome teeth is observed which we interpret as a ‘scooping’ mechanism resulting in an upwards transport of spores. The following outward movement during desiccation can be seen as a mechanism exposing the spores clinging to the teeth structures to the air. Additionally, the whole spore capsule undergoes shape and mass changes during hydration and desiccation. The capsule shrinks during desiccation and a water droplet is ‘squeezed’ through the dome of the endostome, bringing up further spores from the interior of the capsule.

To our knowledge, this study represents the first kinematic analysis of the hygroscopic shape changes of outer peristome teeth in a moss and gives insights into the underlying functional morphology and anatomy. We hypothesize that longitudinal gradients in material composition, structure and geometry of the two layers composing the peristome teeth result (i) in differences of swelling/shrinking capacity and velocity between the two layers composing the teeth, and (ii) in a gradient of swelling velocity and shrinking from the tip to the base of the teeth that provide the structural background of the observed movements. This hypothesis is corroborated by what we believe to be the first experiments using isolated layers of peristome teeth.

## Sources of Funding

S.P. would like to thank the ‘Joint Research Network on Advanced Materials and Systems’ (JONAS) for funding. The article processing charge was funded by the German Research Foundation (DFG) and the University of Freiburg in the funding programme Open Access Publishing.

## Contributions by the Authors

F.G., S.P. and T.S. designed and conceptualized the study. M.L. conducted the experiments and H.-H.K. produced LT-SEM images. F.G., S.P. and T.S. supervised and supported the experimental work and data analysis. F.G. wrote the manuscript, M.L. assisted in drafting the manuscript and all authors revised the manuscript.

## Conflict of Interest

None declared.

## Supporting Information

The following additional information is available in the online version of this article—

**Video 1.** Hygroscopic movements of an outer peristome tooth during hydration (real-time).

**Video 2A.** Hygroscopic movements of an outer peristome tooth during desiccation (phase I) (real-time).

**Video 2B.** Hygroscopic movements of an outer peristome tooth during desiccation (phase II) (real-time).

**Video 3A.** Shape change of a spore capsule of Brachythecium populeum during hydration (time lapse ×20).

**Video 3B.** Shape change of a spore capsule of Brachythecium populeum during desiccation (time lapse × 40).

**Video 4.** Spores sifting through the peristome of a spore capsule of Brachythecium populeum (slow motion × 200).

**Video 5A.** Hydration and desiccation of an isolated inner layer of an exostome tooth of Brachythecium populeum (time lapse ×6).

**Video 5B.** Hydration and desiccation of an isolated outer layer of an exostome tooth of Brachythecium populeum (time lapse ×6).

## Supplementary Material

Supplemental Video1

Supplemental Video2A

Supplemental Video2B

Supplemental Video3A

Supplemental Video3B

Supplemental Video4

Supplemental Video5A

Supplemental Video5B
